# Dynamic Simulation of the Leaf Mass per Area (LMA) in Multilayer Crowns of Young *Larix principis-rupprechtii*

**DOI:** 10.3390/plants13091223

**Published:** 2024-04-28

**Authors:** Jinshan Wang, Ying Zhou, Cuiying Ji, Longfei Xie, Qiang Liu, Zhidong Zhang

**Affiliations:** 1 School of Forestry, Hebei Agriculture University, Baoding 071001, China; 13634754191@163.com (J.W.); zhouy1117@126.com (Y.Z.);; 2 School of Forestry, Beihua University, Jilin 132013, China; xielongfei@beihua.edu.cn

**Keywords:** *Larix principis-rupprechtii*, leaf mass per area, leaf dry matter content, crown whorl, leaf growth phase, predictive models

## Abstract

Leaf mass per area (LMA) is a key structural parameter that reflects the functional traits of leaves and plays a vital role in simulating the material and energy cycles of plant ecosystems. In this study, vertical whorl-by-whorl sampling of LMA was conducted in a young *Larix principis-rupprechtii* plantation during the growing season at the Saihanba Forest Farm. The vertical and seasonal variations in LMA were analysed. Subsequently, a predictive model of LMA was constructed. The results revealed that the LMA varied significantly between different crown whorls and growing periods. In the vertical direction of the crown, the LMA decreased with increasing crown depth, but the range of LMA values from the tree top to the bottom was, on average, 30.4 g/m^2^, which was approximately 2.5 times greater in the fully expanded phase than in the early leaf-expanding phase. During different growing periods, the LMA exhibited an allometric growth trend that increased during the leaf-expanding phase and then tended to stabilize. However, the range of LMA values throughout the growing period was, on average, 40.4 g/m^2^. Among the univariate models, the leaf dry matter content (LDMC) performed well (adjusted determination coefficient (*R*_a_^2^) = 0.45, root mean square error (RMSE) = 13.48 g/m^2^) in estimating the LMA. The correlation between LMA and LDMC significantly differed at different growth stages and at different vertical crown whorls. The dynamic predictive model of LMA constructed with the relative depth in the crown (RDINC) and date of the year (DOY) as independent variables was reliable in both the assessments (*R*_a_^2^ = 0.68, RMSE = 10.25 g/m^2^) and the validation (absolute mean error (MAE) = 8.05 g/m^2^, fit index (FI) = 0.682). Dynamic simulations of crown LMA provide a basis for elucidating the mechanism of crown development and laying the foundation for the construction of an ecological process model.

## 1. Introduction

The leaf economics spectrum (LES) is a set of interconnected and synergistic functional traits that quantitatively represent a range of steadily shifting plant resource trade-off strategies [1,2]. At the heart of this complex and multifaceted trait network lies leaf mass per area (LMA) [3]. LMA is the ratio of dry leaf mass to the corresponding leaf area and is a combination of various leaf anatomical characteristics [4]. It is widely used to estimate leaf area indices [5,6] and simulate canopy photosynthesis [7,8]. LMA values vary among different tree species, different environmental conditions, and different leaf developmental stages. Thus, accurate and swift measurements of LMA and its dynamic changes are highly important for understanding the growth processes of trees, simulating canopy photosynthesis, and estimating forest productivity.

The crown is the primary organ responsible for photosynthesis in trees. Its intricate three-dimensional structure of branches and leaves affects the local microenvironment of the crown, resulting in spatial differences in leaf functional traits in different areas of the crown [9,10,11]. Studies have shown that the LMA of crowns in various forest types tends to increase from the bottom to the top of the crown [12,13,14]. This increasing pattern of LMA is usually associated with the light gradient through the crown and the water potential gradient from the root to the crown [15,16,17,18]. In addition to the vertical variation in the crown, LMA also varies significantly across the different developmental stages of trees. Nouvellon et al. [19] reported that LMA changes drastically over different months, which was also confirmed by Rossatto’s research [20] on savanna grassland tree species and forest tree species in central Brazil. This variation in LMA can be attributed to differences in temperature, precipitation, and solar radiation among the different periods.

The alteration of LMA typically depends on the leaf dry matter content (LDMC). Plants can acclimatize to diverse circumstances through varied dry matter investments. Consequently, the correlation between traits is strongly associated with the resources and environment in which plants are located. Previous studies have demonstrated that there is a significant correlation between LMA and LDMC, and the association is significantly disparate under diverse environmental conditions [21,22]. For trees with a conspicuous canopy structure, the gradient discrepancy of the canopy microenvironment in the vertical direction [23] will cause a shift in the correlation between different canopy depths. Zhang [24] studied the vertical changes in the LMA and LDMC of *Pinus yunnanensis* with canopy height. The results showed that different LMA and LDMC values exhibited distinct changes with canopy height. Tian et al. [25] reached the same conclusion. Additionally, alterations in the temperature, precipitation, and solar radiation of plants during different growing seasons will make the environment in which leaves are situated highly heterogeneous. This will lead to different changes in LMA and LDMC with canopy height at different growth stages.

Due to the limitations of leaf area measurement technology [26], it is very difficult to measure LMA. Currently, LMA is measured by retaining some leaves of the analysed tree and establishing a single tree leaf biomass model based on the leaf biomass of the analysed tree and its diameter at breast height [27], or by directly calculating LMA from the measured total leaf area and leaf dry mass. However, for coniferous plants, these methods require considerable manpower and material resources [28,29] because of their three-dimensional structure and large number of leaves. To address this issue, an increasing number of researchers are estimating LMA by establishing regression models between LMA and plant traits, leaf morphology, or environmental conditions, such as leaf length, leaf width [30,31], branch height [32,33], and LDMC [34,35,36]. As the correlation between LMA and the vertical direction of a tree crown is significant, variables related to vertical height, such as branch height, depth into the crown, and relative depth into the crown, are often used as the main fitting factors. LDMC is also a common fitting factor, and many studies have discussed the relationship between these two parameters. Typical linear models or nonlinear models are used to fit LMA based on LDMC [37]. Peng’s research [38] showed that the LMA of Chinese fir can be estimated by the LDMC and that the model meets the estimation requirements. Therefore, it is important to establish a simple and accurate LMA prediction model for the purpose of simplifying canopy models. Determining LDMC and RDINC is simpler than determining LMA, and both methods meet the estimation requirements of LMA. However, previous studies have taken only leaf samples at one particular point in time or at a specific canopy position. It is yet to be determined whether different leaf development stages and depths of the canopy have an effect on LMA prediction models. A few studies have examined which vertical factor or LDMC can most accurately predict LMA. Furthermore, whether leaf development time can be used as a single factor to predict LMA has not been tested.

*Larix principis-rupprechtii*, one of the most widely planted trees in North China, is characterized by strong light tolerance, rapid growth, and longevity and is a valuable native species. This study aimed to clarify the variation patterns of the LMA of needles in different vertical layers and at various leaf development stages and to reveal the main factors influencing the LMA. Finally, the best prediction model of LMA for *L. principis-rupprechtii* plantations was established, which can be used to simulate crown photosynthesis and estimate regional primary productivity.

## 2. Results

### 2.1. Correlations between LMA and DOY and between RDINC and LDMC

**Figure 1:**
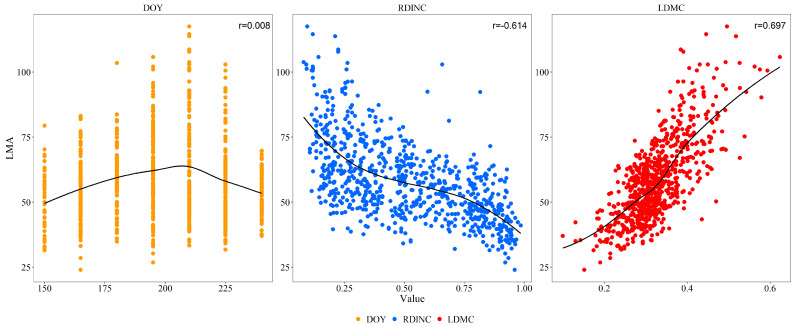
Scatter plot and cor plot. LMA is the leaf mass per area, DOY is the duration of the year, RDINC is the relative depth into the crown, and LDMC is the leaf dry matter content. The solid black lines are trendlines of polynomial equations.

It was evident from the results (Figure 1) that there was a significant negative correlation between LMA and RDINC, with a correlation coefficient of −0.614. However, LMA had a significant positive correlation with LDMC (r = 0.697). LMA had no significant correlation with DOY but showed a unimodal trend with increasing DOY.

### 2.2. Variation in LMA at Different Crown Depths and Leaf Development Stages

**Figure 2:**
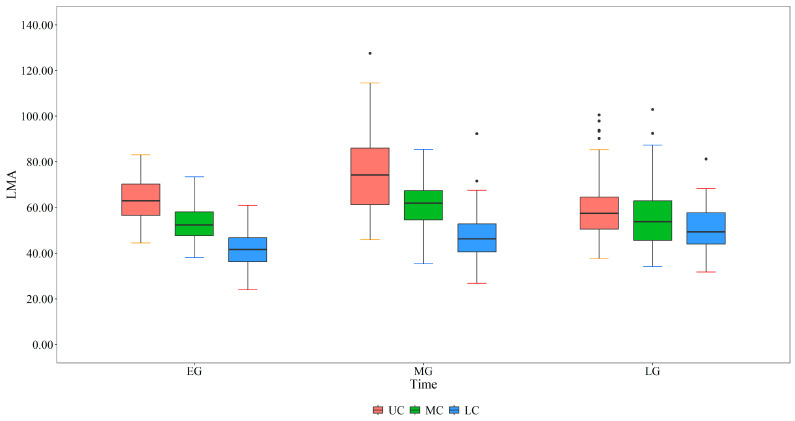
Vertical patterns of leaf mass per area at different leaf growth phases. LMA is the leaf mass per area, UC is the upper crown, MC is the middle crown, and LC is the lower crown.

The results of a two-factor ANOVA (Table 1) revealed that the LMA of *L. principis-rupprechtii* significantly differed among leaf development stages and crown layers, and the interaction between the two factors had a significant effect on the LMA. There were significant differences between the upper, middle, and lower layers of the crown at different stages of leaf development (Figure 2). At the start of leaf development, the LMA decreased from the upper crown (66.68 g·m^−2^) to the lower crown (38.31 g·m^−2^). At the middle stage of leaf development, the LMA ranged from 92.38 g·m^−2^ at the top of the crown to 46.80 g·m^−2^ at the bottom of the crown. At the end of leaf development, the LMA decreased from 68.24 g·m^−2^ at the top of the crown to 47.17 g·m^−2^ at the bottom of the crown.

### 2.3. Correlation Analysis Results

**Figure 3:**
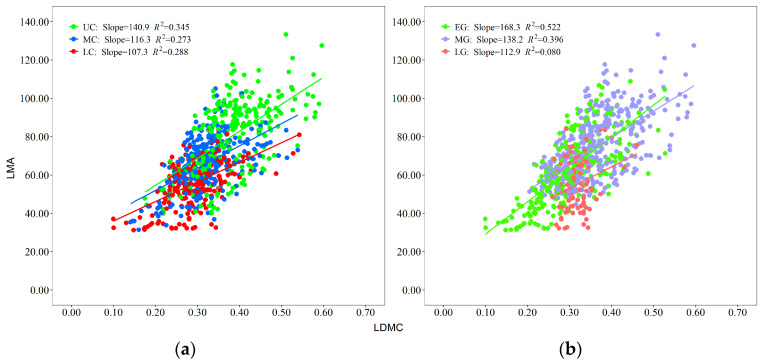
Regression analysis of standard spindles: (**a**) Relationships between leaf mass per area and leaf dry matter concentration in the lower crown (LC), middle crown (MC), and upper crown (UC); (**b**) Relationships between leaf mass per area and leaf dry matter concentration in the early growth phase (EG), middle growth phase (MG), and late growth phase (LG).

There was a significant positive correlation between LMA and LDMC across different crown layers and leaf growth phases (Figure 3). The slopes decreased from the upper crown to the lower crown (Figure 3a), and the slopes decreased from the early growth phase to the late growth phase (Figure 3b). On average, the correlations between LMA and LDMC were greater when grouped by leaf growth phase than when grouped by crown layer.

### 2.4. Model Fitting and Validation Results

Table 2 illustrates the goodness-of-fit results of the 7 basic models that were established with RDINC (Model 1~4), DOY (Model 5), and LDMC (Model 6~7). The models that were established based on DOY and LDMC showed better fitting performance than those that were established based on RDINC, with a high modified determination coefficient (*R*_a_^2^), low root mean square error (RMSE), and Akaike information criterion (AIC). Model 7 showed the best goodness-of-fit, with the highest *R*_a_^2^ value (0.447) and lowest RMSE value (9.38 g·m^−2^).

The method of reparameterization was used to establish bivariate models based on the basic models (Table 2), resulting in Model 8~Model 13 (Table 3). The results showed that the fitting performances of the bivariate models were better than those of the univariate models (Table 2). Model 13 was the only model with an *R*_a_^2^ greater than 0.6 and an RMSE lower than 11 g·m^−2^. The validation result of Model 13 also performed best, with the lowest MAE (MAE = 8.05) and the highest IF (0.682). The MEs of Model 8~Model 13 were all negative, indicating that those models were slightly overestimated.

## 3. Discussion

### 3.1. Temporal and Spatial Variation in LMA

Our results showed that LMA decreased gradually with increasing RDINC. Studies by Marshall and Monserud [32], Burgess and Dawson [33], Zhou et al. [34], and Tian et al. [25] on the vertical variation in crown LMA in *Pinus monticola*, *Sequoiadendron giganteum*, *Betula platyphylla*, and *Platycladus orientalis* have shown that crown structure is the most direct and active interface between plants and their external environment. Complex crown structures usually exhibit different microenvironmental conditions, such as light, temperature, and water vapour deficit pressure [8]. The upper leaves of the crown, which are exposed to strong light radiation and other conditions (e.g., temperature, wind speed, and humidity), increase dry matter input to produce more protective tissues to resist the external environment [39,40]. Additionally, the upward movement of water from roots to crowns inevitably leads to a decrease in the upper water potential [41]. Under water stress conditions, leaves increase investment in vascular tissues and promote water transport to compensate for the impact of lower leaf water potential, resulting in an increase in LMA [42,43]. Conversely, leaves face strong neighbourhood interference and fierce competition for light resources at the bottom of the crown, resulting in a lower LMA with a large and thin morphology.

During the leaf-spreading process, LMA showed a unimodal relationship with DOY (Figure 1) due to an increase in mesophyll cell numbers, inclusions, and cell wall thickness, as well as the maturation of mechanical tissue [15]. May and June are the periods of leaf spread and early leaf growth, and leaves spread quickly to enhance light-capturing abilities, resulting in a lower LMA. During this period, leaves grow rapidly with the division and expansion of leaf cells, which is consistent with the results of Meinzer et al. [44]. LMA significantly increased in the middle growth phase, particularly in the upper crown (Figure 2), which was similar to the finding that the LMA of leaves developing outside the tree crown was significantly greater than that of leaves developing inside the crown [21,45] because of light induction. In addition, plants increase leaf thickness to improve leaf photosynthesis and stress resistance, which leads to an increase in the accumulation of nonstructural carbohydrates in cells [46] and consequently an increase in the LMA. Subsequently, the leaves enter the senescence stage, nutrients are translocated to the branches, the leaves lose water, and the LMA decreases [47]. Using time-integrated irradiance (PPFDINT), Colbe et al. [48] elucidated the seasonal increase in LMA in sugar maple leaves and demonstrated that the leaves are a long-term adaptation to light, with both seasonal accumulation and light intensity having significant impacts on LMA.

### 3.2. Changes in the Correlations between LMA and LDMC in Various Crown Layers (CLs) and Leaf Growth Phases (LGPs)

LMA and LDMC are interdependent leaf features. The distinct LMA and LDMC distributions revealed two plant resource allocation strategies, namely, accelerated expansion of the light absorption capacity and efficient storage of materials for leaf structure formation [49,50]. Leaf LMA and LDMC not only vary in a wide range of environments but also respond to different situations caused by alterations in crown depth or the leaf growth phase [51,52]. Our study revealed that there was a positive correlation between LMA and LDMC, but the patterns of their correlations differed among different CLs and LGPs (Figure 3). The increase rate of LMA with LDMC decreased from the upper crown layer to the lower crown layer (Figure 3a), implying that plants allocate more dry matter to the highest region of the crown for the same unit of fresh foliage area [53]. Leaves in the upper crown exposed to intense light may face an environment with increased investment in water transportation and relatively less availability of water [54]. Thus, leaves increase dry matter input and vascular tissue investment to improve their competitiveness and survival [13]. A denser cell structure and narrower air space could accelerate the exchange rate of leaf water, nutrients, etc., thus optimizing photosynthetic income and resulting in a greater LDMC. On the other hand, the light in the lower crown was weak, and the leaves should adapt to weak light and relatively low dry matter input, resulting in a larger leaf area [55]. This was in line with the research results of Tobias et al. [56].

As the development process of leaves increased, the slopes of the relationships between LMA and LDMC decreased (Figure 3b). The lowest LMA (31.25 g/m^2^) and LDMC (0.10 g) were observed in the early leaf growth phase (May–June). During this period, leaves tended to allocate less LMA and lower LDMC to enhance the capacity for capturing light, which was beneficial for competition [53]. During the middle leaf growth phase (July–August), the leaves had the highest values of LDMC (0.60 g) and LMA (133.33 g/m^2^). During this period, the leaves were fully mature, displaying the strongest photosynthetic activity and dry matter production capability [14,57]. This finding was consistent with the research of Liu et al. [58], who showed that the crown of *Larix olgensis* had the greatest photosynthetic capacity in July and August. During the late leaf growth phase, the photosynthetic ability and carbon assimilation rate decrease to a certain extent with low temperatures and nutrient depletion, leading to decreases in LDMC and LMA [19,59].

### 3.3. Optimal Model Selection

The vertical indices of crowns are the most commonly used leaf functional indicators [12,32]. Previous studies have shown that linear, power, and exponential models, which were constructed based on RDINC, achieved good fitting performance. However, Peng et al. [38] demonstrated that the model using LDMC as a separate element performed well, which was consistent with our results (Table 2). The *R*_a_^2^ values for Models 6 and 7 exceeded 0.44, and the RMSEs were lower than 14.0 g·m^−2^. Fewer previous studies have considered the influence of different leaf growth phases, especially the early and later leaf growth phases, on the accuracy of the model. Coble et al. [60] demonstrated that disregarding seasonal factors would cause a biassed estimation of LMA. Bivariate modelling improved the goodness-of-fit (Table 3). After reparameterization, the accuracy of Model 8~Model 13 significantly improved, and the *R*_a_^2^ value increased by more than 0.2 compared to that of the univariate model. Model 13 was chosen as the optimal model with the highest *R*_a_^2^ (0.678) and lowest RMSE (10.25 g/m^2^). Interestingly, LDMC, which had the highest correlation with LMA, was not included in the optimal model (Model 13). Fortunately, model 13 had the ability to dynamically predict the DOY.

## 4. Materials and Methods

### 4.1. Site Description

The study site was located at the Saihanba Forest Farm, Hebei Province, northern China (42°02′~42°36′ N, 116°51′~117°39′ E), at an altitude of 1010~1939.9 m. The main soil type is sand. The climate type is a typical temperate continental monsoon climate, with an annual average temperature of −1.3 °C, an extreme minimum temperature of −43.3 °C, an extreme maximum temperature of 33.4 °C, an annual average snow cover of 7 months, an annual average precipitation of 460 mm, an average annual frost-free period of 64 days, and an annual average windy day of 53 days. The main tree species are *L. principis-rupprechtii*, *Picea asperata*, *Betula platyphylla*, *P. sylvestris* var. *Mongolica sylvestris*, etc. The forest coverage rate was 82%, and the total forest stock was 5.025 million m^3^.

### 4.2. Sample Selection

In this research, five sample plots (20 m × 30 m) were established within a 17-year-old, pure *L. principis-rupprechtii* plantation in the same habitat as the Saihanba Forest Farm. All trees with a diameter at breast height (DBH) larger than 5 cm in the sample plot were measured, and factors such as DBH, tree height (H), crown width (CW), and relative coordinates (X,Y) were included. Subsequently, five sample trees with a DBH similar to the quadratic mean diameter (Dg), representing the average state of each sample plot, were chosen. The basic information about the sample plots and sample trees is displayed in Table 4.

### 4.3. Measurement of LMA

For a single tree, the crown was divided into various whorls by the whorls of branches from top to bottom. In each group, 3–4 healthy clusters were chosen as samples. The relative depth into the crown (the ratio of depth into the crown to crown length, RDINC) of every sample cluster was recorded, and then the samples were immediately removed and taken back to the laboratory for scanning and weighed immediately to determine fresh weight (WF). The scanned needle samples were dried to a constant weight of 85 °C and weighed (WD). The images were analysed using image analysis software (Image-Pro Plus 6.0, Media Cybernetics, Inc., Bethesda, MD, USA), resulting in a projected leaf area (LA, m^2^). The LMA and LDMC of each cluster of needle samples were then calculated. The data were collected every half month during the growing phases (approximately from 1 June to 15 September) in 2017. The basic statistics were listed in Table 5.

The LMA and LDMC of each cluster of needle samples were calculated as follows:

LMA_i,j_ = WD_i,j_/LA_i,j_ (1)

LDMC_i,j_ = WF_i,j_/WD_i,j_ (2)

where _i_ represents the sample whorls, _j_ represents the date of the measurement, WD represents the dry weight, and WF represents the fresh weight.

### 4.4. Model Descriptions

#### 4.4.1. Basic Model Selection

Based on previous research and the scatter plot distribution and correlation between LMA and LDMC, as well as the spatial position and leaf growth phase (LGP) obtained in this study, a basic model was established with RDINC, date of year (DOY), and LDMC as independent variables (see Table 6).

#### 4.4.2. Discrete Analysis and Reparameterization

According to prior research, the relationships between LMA and LDMC, leaf spatial position, and leaf growth phase are evident. Thus, to improve the accuracy of the model, it is necessary to further discretize the LMA data for feature analysis. Models 1–4 simulated the LMA in 9 groups, with intervals of 0.1, based on RDINC. Model 5 simulated the LMA in 6 groups, with groupings of 150, 165, 180, 195, 210, and 225, based on the DOY. Models 6–7 simulated the LMA on RDINC and DOY. Then, reparameterization was conducted in Models 1–7, according to the correlations between the parameters and RDINC and DOY, to form 6 new models with multiple independent variables (RDINC, DOY, and LDMC). Finally, the optimal LMA model for *L. principis-rupprechtii* was selected based on its goodness-of-fit (Equations (3)–(5)) and validation performance (Equations (6)–(8)). The LMA prediction model was then established through parameterization.

#### 4.4.3. Model Assessment and Validation

When fitting the model, 75% of the data were randomly chosen for model fitting, and 25% were used for model validation (Table 7). The indicators chosen to assess the model’s goodness-of-fit are the adjusted determination coefficient (*R*_a_^2^), root mean square error (RMSE), and Akaike information criterion (AIC). The indicators for validation are the mean error (ME), absolute mean error (MAE), and fit index (FI). The formulas for computing each index are as follows:

(3) $$R_{a}^{2}=1-\left(1-R^{2}\right)\left(\frac{n-1}{n-p}\right)a=1,\;\mathrm{where}\;R^{2}=1-\frac{\sum_{i=1}^{n}{\left(y_{i}-{\hat{y}}_{i}\right)}^{2}}{{\sum_{i=1}^{n}\left(y_{i}-{\bar{y}}_{i}\right)}^{2}}$$

(4) $$RMSE=\sqrt{\frac{\sum_{i=1}^{n}{\left(y_{i}-{\hat{y}}_{i}\right)}^{2}}{n-p}}$$

(5) $$AIC=nIn({\left(y_{i}-{\hat{y}}_{i}\right)}^{2})-nIn(n)+2p$$

(6) $$ME=\sum_{i=1}^{n}\left(\frac{y_{i}-{\hat{y}}_{i}}{n}\right)$$

(7) $$MAE=\sum_{i=1}^{n}\left|\frac{y_{i}-{\hat{y}}_{i}}{n}\right|$$

(8) $$\mathrm{FI}=1-\frac{\sum_{i=1}^{n}{\left(y_{i}-{\hat{y}}_{i}\right)}^{2}}{{\sum_{i=1}^{n}\left(y_{i}-{\bar{y}}_{i}\right)}^{2}}$$

where yi is the observed value, $\bar{y}$i is the average of the observed values, ŷi is the predicted value, n is the number of samples, and *p* is the number of parameters.

### 4.5. Data Analysis

A two-factor ANOVA was used to examine whether the LMA of *L. principis-rupprechtii* significantly differed between different trees and different ring whorls at various growth and developmental stages. Furthermore, Pearson correlation coefficients among LMA, LDMC, RDINC, and DOY were computed, and the correlations between LMA and other factors were analysed. The LMA and LDMC of different canopies and different growth periods were fitted by standardized principal axis analysis. Tests were conducted to determine whether there was a significant difference in slope among the different groups and to ascertain whether different canopy depths and growth periods had a significant influence on the correlation between LMA and LDMC.

Microsoft Excel 2010 was used to collate the data of the study; descriptive statistical analysis was performed using SPSS 24; model fitting was completed by the nls package in R 4.0.5; standardized principal axis analysis was completed by the smatr package in R 4.0.5; and diagrams were drawn with the ggplot2 package in R 4.0.5 and Origin 2019.

## 5. Conclusions

Tree leaves (such as *L. principis-rupprechtii*) can adapt to complex crown structures by altering their own morphological characteristics, which results in significant spatial heterogeneity within the tree crown. In addition, the morphological characteristics of leaves from different crown layers exhibited different seasonal patterns (such as LMA and LDMC). The LMA prediction model using the reparameterization method (Model 13) had the best fitting performance (R^2^ = 0.68, RMSE = 10.25 g/m^2^), and the best validation result was obtained (MAE = 8.05 g/m^2^, FI = 0.682). Furthermore, Model 13 exhibited dynamic predictions that benefited from the incorporation of DOY. The LMA prediction model provides convenience for the rectification of physiological ecological models and provides a reference for branch pruning that considers leaf functional traits.

## Figures and Tables

**Table 1:** Two-way ANOVA of the LMA of *L. principis-rupprechtii*.

Variables	Statistics		
	*df*	*F*	Sig.
Intercept	1	1203.943	<0.001
Leaf growth phase	5	15.929	<0.001
Crown layer	2	57.877	<0.001
Leaf growth phase × Crown layer	10	10.645	<0.001

**Table 2:** The goodness-of-fit results of different basic models.

No.	Model Form	*R*_a_^2^	RMSE	AIC
Model 1	LMA = −38.47 × RDINC + 87.60	0.297	15.194	6452.82
Model 2	LMA = 90.53 × exp(−0.58 × RDINC)	0.302	15.143	6447.61
Model 3	LMA = 27.98 × RDINC^2^ − 68.36 × RDINC + 93.72	0.304	15.116	6446.85
Model 4	LMA = 55.33 × RDINC^−0.24^	0.299	15.171	6450.54
Model 5	LMA = −22.92 + 2.62 × (DOY − 120) + 0.02 × (DOY − 120)^2^	0.403	14.002	6327.61
Model 6	LMA = 156.76 × LDMC + 15.56	0.443	13.524	6271.50
Model 7	LMA = 157.82 × LDMC^0.76^	0.447	13.479	6266.29

**Table 3:** Results of model fitting and validation.

No.	Model From	Goodness-of-Fit			Validation		
		*R*_a_^2^	RMSE	AIC	ME	MAE	IF
Model 8	LMA = (−1.32 × (DOY−120) + 258.30) × LDMC + 0.31 × (DOY − 120) − 7.52	0.470	13.16	4682.5	−0.092	10.753	0.474
Model 9	LMA = (−62.38 × RDINC + 150.84) × LDMC + 27.67	0.507	12.69	4638.0	−0.106	10.176	0.514
Model 10	LMA = (−41.15 × RDINC + 150.95) × LDMC^0.59^	0.515	12.59	4629.0	−0.099	10.040	0.523
Model 11	LMA = (0.0003 × (DOY − 120)^3^ − 0.114 × (DOY − 120)^2^ + 11.38 × (DOY − 120) − 217.1) × LDMC + 32.28	0.598	11.46	4522.8	−0.236	9.353	0.615
Model 12	LMA = (−0.0215 × (DOY − 120)^2^ + 3.256 × (DOY − 120) + 20.264) × LDMC^0.574^	0.593	11.54	4528.3	−0.205	9.318	0.611
Model 13	LMA = (−37.08 × RDINC − 0.44) + (0.003 × RDINC + 2.524) × (DOY − 120) − 0.016 × (DOY − 120)^2^	0.678	10.25	4390.5	−0.154	8.050	0.682

Note: LMA represents leaf mass per area, RDINC represents relative depth into the crown, DOY represents date of year, and LDMC represents the leaf dry matter content.

**Table 4:** The attributes of the sample plots and the sample trees were outlined.

Sample Plots					Sample Trees		
Plot Number	Age (Year)	Quadratic Mean Diameter (cm)	Mean Tree Height (m)	Stand Density (Trees·hm^−2^)	Tree Number	Diameter at Breast Height (cm)	Tree Height (m)
P_1_	17	12.0	11.95	2489	I	11.2	10.5
P_2_	15	10.0	9.95	2461	II	11.0	11.3
P_3_	16	13.5	13.80	2383	III	11.7	10.8
P_4_	16	12.7	12.81	2112	IV	11.2	11.5
P_5_	17	11.9	12.98	2049	V	11.9	10.9

Note: P_1_~P_5_ represent sample plot 1 to sample plot 5.

**Table 5:** The attributes of LMA and LDMC.

Layer	Growth Phase	LMA(g/m^2^)				LDMC(g)			
		Mean	S.D.	Max.	Min.	Mean	S.D.	Max.	Min.
UC	EG	74.07	16.98	108.84	39.88	0.334	0.072	0.526	0.175
	MG	87.62	16.49	133.33	49.39	0.419	0.066	0.596	0.312
	LG	60.52	14.86	84.00	40.00	0.332	0.038	0.430	0.260
MC	EG	63.23	16.72	94.86	31.37	0.291	0.070	0.511	0.141
	MG	70.65	10.33	105.04	48.28	0.343	0.056	0.538	0.233
	LG	56.86	11.78	78.70	36.86	0.317	0.032	0.460	0.263
LC	EG	52.83	14.23	78.08	31.25	0.288	0.085	0.527	0.100
	MG	59.44	7.48	84.29	44.70	0.293	0.044	0.542	0.204
	LG	46.55	10.15	61.03	32.10	0.304	0.024	0.353	0.263
Pooled		67.75	18.12	133.33	31.25	0.333	0.077	0.596	0.099

Note: UC, MC, and LC represent the upper, middle, and lower canopies, respectively, while EG, MG, and LG represent the early growth phase (1 June 1–15 July), middle growth phase (16 July–31 August), and late growth phase (1 September–defoliation), respectively. The same applies below.

**Table 6:** Model forms.

No.	Model Form	Parameter
Model 1	LMA = a_0_ × RDINC + a_1_	a_0_,a_1_
Model 2	LMA = b_0_ × exp(b_1_ × RDINC)	b_0_,b_1_
Model 3	LMA = c_0_ × RDINC^2^ + c_1_ × RDINC + c_2_	c_0_,c_1_,c_2_
Model 4	LMA = d_0_ × RDINC^d1^	d_0_,d_1_
Model 5	LMA = e_0_ × (DOY − 120)_2_ + e_1_ × (DOY − 120) + e_2_	e_0_,e_1_,e_2_
Model 6	LMA = f_0_ × LDMC + f_1_	f_0_,f_1_
Model 7	LMA = g_0_ × exp(g_1_ × LDMC)	g_0_,g_1_

Note: LMA represents leaf mass per area, RDINC represents relative depth into the crown, and DOY represents date of year. Leaves started to germinate when DOY = 120; thus, DOY−120 represents the initial development time of the leaves.

**Table 7:** Statistical summary of fitting data and validation data.

Variables	Fitting Data (No. = 585)				Validation Data (No. = 194)			
	Max.	Min.	S.D.	Mean	Max.	Min.	S.D.	Mean
LMA	133.33	31.25	18.08	67.69	127.53	31.50	18.30	67.93
DOY	240	150	26.88	199.15	240	150	26.57	199.25
LDMC	0.58	0.10	0.08	0.33	0.60	0.10	0.08	0.33
RDINC	0.99	0.08	0.26	0.52	0.96	0.09	0.25	0.51

Note: LMA represents leaf mass per area, RDINC represents relative depth into the crown, DOY represents date of year, and LDMC represents the leaf dry matter content. Max. is the maximum value, Min. is the minimum value, S.D. is the standard deviation, and Mean is the mean value.

## Data Availability

Data are available upon request to the corresponding authors.
